# Effect of packaging materials on the chemical composition and microbiological quality of edible mushroom (Pleurotus ostreatus) grown on cassava peels

**DOI:** 10.1002/fsn3.216

**Published:** 2015-03-12

**Authors:** Oluwakemi Ajayi, Adewale Obadina, Micheal Idowu, Mojisola Adegunwa, Olatundun Kajihausa, Lateef Sanni, Yemisi Asagbra, Bolanle Ashiru, Keith Tomlins

**Affiliations:** 1 Department of Food Science & Technology, Federal University of AgricultureAbeokuta, Nigeria; 2 Department of Hospitality & Tourism, Federal University of AgricultureAbeokuta, Nigeria; 3 Federal Institute of Industrial ResearchOshodi, Ikeja, Lagos P.M.B. 21023, Nigeria; 4 Natural Resources Institute, University of GreenwichMedway Campus, Central Avenue, Chatham Maritime, Kent ME4 4TB, UK

**Keywords:** Freezing temperature, microbial count, mushroom, packaging, proximate compositionproximate composition

## Abstract

Edible fungi such as mushrooms are highly perishable and deteriorate few days after harvest due to its high moisture content and inability to maintain their physiological status. In this study, the effect of packaging materials on the nutritional composition of mushroom cultivated from cassava peels was investigated. Mushroom samples were dried at 50°C in a cabinet dryer for 8 h. The dried mushroom samples packaged in four different packaging materials; high density polyethylene (HDPE), polypropylene (PP), laminated aluminum foil (LAF), high density polyethylene under vacuum (HDPEV) were stored at freezing (0°C) temperatures for 12 weeks. Samples were collected at 2-week intervals and analyzed for proximate composition (carbohydrate, protein, fat, fiber, ash, moisture), mineral content (calcium, potassium), vitamin C content, and microbiological qualities (total aerobic count, *Pseudomonal* count, Coliform count, *Staphylococcal* count, *Salmonella* count) using the standard laboratory procedures. Carbohydrate, protein, fat content of dried mushrooms packaged in HDPE at freezing temperature ranged from 45.2% to 53.5%, 18.0% to 20.3%, and 3.2% to 4.3%, while mushrooms in polypropylene ranged from 45.2% to 53.5%, 18.5% to 20.3%, 2.6% to 4.3%. Carbohydrate, protein, fat of mushroom in LAF ranged from 47.8% to 53.5%, 17.3% to 20.3%, and 3.3% to 4.3%, respectively, while carbohydrate, protein, fat of mushroom in HDPEV ranged from 51.1% to 53.5%, 19.5% to 20.3%, and 3.5% to 4.3%. Microbiological analysis showed that total aerobic count, *Pseudomonal* count, and *Staphyloccocal* count of dried mushroom ranged from 2.3 to 3.8 log cfu/g, 0.6 to 1.1 log cfu/g, and 0.4 to 0.5 log cfu/g, respectively. In conclusion, dried mushroom in HDPE packaged under vacuum at freezing temperature retained the nutritional constituents than those packaged with other packaging materials.

## Introduction

Cassava *Manihot esculenta* is a perennial woody shrub with edible root and leaves, which grows in the tropical and subtropical areas of the world. Cassava originated from tropical America and was first introduced into Africa in the Congo basin by the Portuguese around 1558 (Al-Ati 2003). Today, it is a dietary staple in tropical Africa (Chang and Miles 2004). It is rich in protein, carbohydrate, vitamins B and C, calcium, and essential minerals (Lopez-Briones 1992). The peels of cassava are regarded as solid wastes and are usually discarded and these wastes are generally considered to contribute significantly to environmental pollution and esthetic nuisance (Lee and Sharma 2005). Cassava wastes transformation offers the possibility of creating marketable value-added products such as in the production of edible fungi from the abundantly available agro-industrial waste. After one day of storage at ambient temperature, cap is opened and colored, stem is elongated, and texture becomes soft and spongy, resulting in a depression of its commercial value (Barron 2006). Mushroom species are categorized under three groups namely: Edible mushroom, medicinal mushroom, and poisonous mushroom (Mane and Dawson 2007). One way to preserve mushroom so that they can be consumed longer is by drying (Al-Ati 2003). Fresh mushroom, due to its thin and porous epidermal structure, have relatively high respiration rate because of its high moisture content results in changes in the color, taste, and texture with its shelf life being <3 days under usual transportation and marketing conditions (Kim et al., 2006). Effort has continuously been geared toward reducing environmental pollution through the utilization of agricultural wastes. One of the areas in which this strategy has been applied is the use of waste generated from cassava as a substrate for mushroom cultivation. The success of this technology has been demonstrated for the production of edible mushrooms on commercial basis but the effect of such substrate, packaging material used, and storage conditions on the microbiological safety and quality of edible mushroom are yet to be documented.

Mushroom production in Nigeria has not significantly improved above cottage level. This is because of the seasonality of mushroom production as well as the relative short life span of mushroom which discourages large scale production of mushroom. Therefore, various adaptable techniques other than the conventional ones need to be employed in order to increase the production and mushroom availability throughout the year thereby limiting the importation of mushroom for domestic use.

Therefore, to retain freshness, optimum packaging material that will promote the nutritional composition of mushroom while preserving quality and safety is required. This research is aimed at identifying the packaging materials that are suitable for extending the shelf life of dried mushroom produced from cassava peels.

## Materials and Methods

Cassava peels were obtained from Isolu Community, along Alabata road, Abeokuta, Ogun State, after which the periderm was detached from the cortex, this was done in order to rid the peel of the sugar present. The peels were then sun dried and transported to the Federal Institute of Industrial Research, Oshodi, Nigeria where mushroom cultivation was carried out while packaging materials were obtained from Ojota, Lagos.

The dried cassava peels were crushed and mixed with water and calcium carbonate at a ratio of 32:66:2 to make them moist. The substrate was squeezed to ensure that they have the appropriate moisture content (a squeeze of the substrate should give 2–3 water drops, moisture content at this point should be 70%). The compost was continuously turned for 3 days after which they were dispensed into polythene bags and autoclaved at 121°C for 2 h to rid them of microbes that might contaminate the material during the process or use the available nutrients necessary for the mushroom growth. Spawn of 2.5% dry weight of the substrates was aseptically added to the substrate after cooling. The bags were hermetically sealed and punctured at the base for minimum aeration. The inoculated substrates were placed in a mushroom house which had a relative humidity of about 65–70% and monitored daily. They were monitored daily until the substrate was completely ramified with white mycelia. The ramified substrates were exposed to air by loosening the polythene bag to allow air circulation and light penetration till the pin heads appeared. After the appearance of the pin heads the fruiting bodies of the mushroom completely appeared and were carefully picked from the substrate. Mushrooms from different flushes and phases were continuously harvested from the substrate and fresh healthy mushrooms were dried using cabinet drier and allowed to cool.

Twenty grams of mushroom sample was packaged in four different packaging materials (bags) which include high density polyethylene, polypropylene, laminated aluminum foil, and in high density polyethylene packaged under vacuum which were stored at freezing temperature for 12 weeks. Subsamples of mushroom were collected every 2 weeks until 12 weeks of age for chemical analyses and safety assessments. All experiments were conducted in triplicates.

### Chemical analyses

The chemical analyses were conducted using AOAC (2000).

### Determination of crude protein content

Dried mushroom sample (0.5 g), 1 Kjeldahl catalyst tablet, and 10 mL of concentrated H_2_SO_4_ were added into a micro-Kjeldahl flask. The digestion was left on for 4 h until a clear colorless solution was left in the digestion tube. The digested product was carefully transferred into 100-mL volumetric flask, thoroughly rinsing the digestion tube with distilled water and the volume of the flask made up using distilled water. It was then pipetted into a distillation apparatus and 5 mL of 40% (w/v) NaOH was added. The mixture was then steam distilled and the liberated ammonia was collected in a 50 mL conical flask containing 10 mL of 2% boric acid and indicator solution. The green color solution was then titrated against 0.01 mol/L HCl solution.

### Determination of crude fat content

One gram of dried mushroom sample was weighed into a fat-free extraction thimble and plugged lightly with cotton wool. The thimble was placed in the extractor and fitted with reflux condenser and a 250-mL soxhlet flask was cooled in the desiccator and weighed. The soxhlet flask was then filled to three quarter of its volume using n-hexane and the soxhlet flask extractor plus condenser set were placed on the heater. The heater was switched on for 6 h with constant running water from the tap for condensation of ether vapor. The ether was left to siphon over several times (at least 10–12 times) until it was short of siphoning. The thimble-containing sample was then removed and dried on a clock glass on the bench top. The extractor flask with condenser was replaced and the distillation continues until the flask was practically dried out. The flask which now contains the fat or oil was detached, its exterior cleaned and dried to a constant weight in the oven.

The percentage fat content was calculated as follows: 

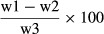


w1 = initial weight of dry soxhlet flask; w2 = and the final weight of oven-dried flask + oil; w3 = weight of sample taken.

### Determination of fiber content

Two grams of the dried mushroom sample was accurately weighed into the fiber flask and 100 mL of 0.255 N H_2_SO_4_ was added. The mixture was heated under reflux for 1 h with the heating mantle. The hot mixture was filtered through a fiber sieve cloth. The filtrate obtained was discarded and the residue returned to the fiber flask to which 100 mL of 0.313 N NaOH was added and heated under reflux for another hour. The mixture was filtered through a fiber sieve cloth and 10 mL of acetone added to dissolve any organic constituent. The residue was washed with about 50 mL of hot water on the sieve cloth before it was finally transferred into a crucible. The crucible and the residue were oven-dried at 105°C overnight to drive off any moisture residue. The oven-dried crucible containing the residue was cooled in a desiccator and later weighed to obtain the weight W1. The crucible with weight W1 was transferred to the muffle furnace for ashing at 550°C for 4 h. The crucible containing white or gray ash (free of carbonaceous material) was cooled in the desiccator and weight to obtain W2. The percentage fiber was obtained by the formula: 

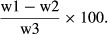


### Determination of total ash content

Two grams of mushroom sample was placed in a clean dry preweighed crucible, and then the crucible with its content ignited in a muffle furnace at about 550°C for 6 h until light gray ash was obtained. The crucible was removed from the furnace to a desiccator to cool and then weighed. The crucible was reignited in the furnace and allowed to cooling until a constant weight was obtained (w2). Total ash content was calculated using the following: 

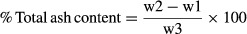


w1 = weight of empty crucible, w2 = weight of crucible with ash, w3 = weight of sample.

### Determination of moisture content

Two grams of mushroom sample was weighed in a clean dry and preweighed crucible and then placed in an oven at 105°C for 3 h. The crucible was transferred to a desiccator and allowed to cool and then weighed. Further placement in the oven was carried out until a constant weight was obtained (w3). Moisture content was calculated using the following formula: 

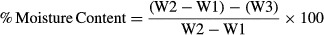


where: W1 = weight of empty crucible, W2 = weight of crucible with the sample, W3 = weight after drying.

### Determination of mineral content

Calcium was measured using Atomic Absorption Spectrometer. Prior to digestion, 0.50 g of mushroom sample was weighed into a 125 mL Erlenmeyer flask with the addition of perchloric acid (4 mL), concentrated HNO_3_, and concentrated sulfuric acid (2 mL) under a fume hood. The contents were mixed and gently heated in a digester under a low to medium heat on a hot plate under perchloric acid fume hood until dense white fumes appeared. The temperature during heating was increased for half a minute, then allowed to cool, and then 50 mL of distilled water was added. It was filtered completely into a Pyrex volumetric flask and then made up with distilled water and absorbance was read using an Atomic absorption spectrometer (422.7 nm).

### Determination of potassium content

The mushroom samples were first oven-dried at 60°C for 24 h. The dried samples were powdered in a mixer grinder taking care not to overheat the sample. About 0.5 g of dried samples were digested with HNO_3_ and HClO_4_ in a ratio 5:1 until a transparent solution was obtained. The digests were filtered and diluted to 25 mL of distilled water. Analytical reagent grade chemicals were used. Flame Photometer was used to determine potassium after digestion following the standard methods.

### Determination of Vitamin C

Preparation of Metaphosphoric Acetic Acid Solution (MPAA): To 15 g of metaphosphoric acid, 40 mL of glacial acetic acid was added and diluted with 100 mL of water.

Preparation of 2, 6-Dichlorophenol Indophenol Solution: 2, 6-dichlorophenol indophenols salt (0.05 g) was diluted with 100 mL of water and the solution was filtered.

Preparation of Standard Solution Stock Solution: To 0.05 g of L-ascorbic acid standard, 20 mL of MPAA solution was added and diluted with 250 mL water.

Preparation of Sample Solution: To 10 g of mushroom powder, 20 mL of MPAA solution was added and then it was diluted with 500 mL water. Subsequently the solution was filtered through filter paper. To 10 mL of standard stock solution, 5 mL of MPAA solution was added and titrated against 2, 6-dichloro-phenole indophenol solution till the appearance and persistence of pink color for 10 sec. The titration was completed within 2 min and was noted. 

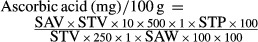


where SAV refers to sample titer value; STV refers to standard titer value; STW refers to standard weigh, SAW refers to sample weight, and STP refers to standard purity.

### Determination of carbohydrate content

The carbohydrate content was determined by subtracting total sum of the % moisture, protein, fiber, fat, and ash from 100.

### Microbiological analysis

One gram (1 g) of *Pleurotus ostreatus* was homogenized in 9.0 mL sterile 0.1% peptone water for 30 sec (normal speed) to a homogenous suspension and then a 10-fold serial dilution in peptone water was carried out (Harrigan 1998).

### Total aerobic count

For total aerobic bacteria count, 1 mL aliquot was plated on Nutrient agar (CM003, Oxoid Ltd, Basingstoke Hants, UK) plates and the plates were incubated at 37°C for 24 h.

### Pseudomonal count

For *Pseudomonas* spp. count, 1 mL of aliquot was plated on pseudomonas agar (CM0559, Oxoid Ltd) with CFC selective agar supplement (SR0103, Oxoid Ltd). Plates were incubated at 25°C and plates examined at 48 h.

### Coliform count

For total coliform count (TCC), 1 mL of aliquot was plated on MacConkey agar (DM 143, Micro master, Maharajahs, India). Plates were incubated at 37°C and plates examined at 48 h.

### Staphylococcal count

For *Staphylococcus* spp. count, 1 mL of aliquot was plated on Mannitol salt agar (CM 0085, Oxoid) supplemented with 5% egg yolk. Plates were incubated at 25°C and plates examined at 24 h.

### Salmonella count

For *Salmonella* spp. count, 1 mL of aliquot was plated on Salmonella Shigella agar. Plates were incubated at 25°C and plates examined at 48 h.

### Statistical analysis

All procedures were carried out in triplicates and data collected from the study were subjected to an analysis of variance (ANOVA). Differences among means were separated using Duncan's Multiple Range Test and significant differences were considered when *P* < 0.05.

## Results and Discussion

The changes in the nutritional composition in high density polyethylene at freezing temperature are shown in Table1. The carbohydrate, protein, and crude fat content reduced significantly from 53.50% to 45.21%, 20.28% to 18.01%, and 4.34% to 3.21%, respectively, while the moisture content increases from 4.38% to 12.21%. The changes in mushroom packaged in polypropylene at freezing temperature are shown in Table2. Carbohydrate, Protein, and fat reduced throughout the storage period from 53.50% to 45.24%, 20.28% to 18.54%, and 4.34% to 2.64%, respectively, while moisture content increases significantly from 4.38% to 12.54%. Ash and fiber showed no significant difference as they increased from 13.49% to 13.43% and 7.67% to 7.61%. The changes in mushroom in laminated aluminum foil at freezing temperature are shown in Table3. Carbohydrate, protein, fat reduced from 53.50% to 47.01%, 20.28% to 17.34%, and 4.34% to 3.32%, respectively. Fiber and ash reduced slightly from 13.49% to 13.36% and from 7.67% to 7.55%, while moisture content increased from 4.38% to 11.42%. Calcium and vitamin C reduced from 54.01 to 49.99 mg/100 g and 42.80 to 23.74 mg/100 g. The change in the nutritional composition of dried mushroom packaged in HDPE and packaged under vacuum at freezing temperature is shown in Table4. Carbohydrate, protein, fat reduced slightly from 53.50% to 48.50%, 20.28% to 19.14% and from 4.34% to 3.42% while fiber and ash also reduced insignificantly from 13.49% to 13.30% and from 7.67% to 7.59% while moisture content increased from 4.38% to 6.64%. The microbial assessment of dried mushroom in different packaging materials is shown in Table5. Dried mushroom packaged in HDPE at freezing temperature showed the count 3.24 and 1.13 for *total aerobic count* and *Pseudomonal count*. Dried mushroom in laminated aluminum foil at freezing temperature showed the count of 2.75, 1.54, and 0.47 log cfu/g for total aerobic count*, Pseudomonal count and Staphylococcal count* while vacuum packaged dried mushroom at freezing temperature gave the count 2.31, 0.46, and 0.37 log cfu/g for *total aerobic count, Pseudomonas*, *and Staphylococcal* count. *Salmonella* and *Coliform* spp. were not detected in all the samples analyzed.

### Effect of HDPE at freezing temperature on the nutritional composition

The changes in the nutritional composition in high density polyethylene at freezing temperature are shown in Table1. The carbohydrate, crude protein, and crude fat content reduced significantly (*P* < 0.05) from 53.5% to 45.2%, 20.3% to 18.0%, and 4.3% to 3.2%, respectively, while the moisture content increases from 4.4% to 12.2%. Mushroom packaged in HDPE bags at freezing temperature also significantly reduced (*P* < 0.05) the carbohydrate, crude protein, and fat composition. This reduction may be attributed to the perforated packaging materials used that allowed for exchange of gases and moisture, enabling the environment favorable for microbes that require these nutrients for various activities (Lin and Durance 1998).

**Table 1:** Proximate composition (%), minerals, and vitamin (mg/100 g) composition of mushroom in HDPE at freezing temperature

Weeks	CHO	Crude protein	Crude fat	Crude fiber	Ash	Moisture	Ca	K	Vitamin C
0	53.5 ± 0.12^a^	20.28 ± 0.05^a^	4.34 ± 0.14^a^	13.49 ± 0.12	7.67 ± 0.02	4.38 ± 0.02^d^	54.01 ± 0.14^a^	3210 ± 0.60	42.80 ± 0.22^a^
2	49.51 ± 0.10^b^	19.61 ± 0.14^b^	4.30 ± 0.08^b^	13.36 ± 0.03	7.58 ± 0.01	5.64 ± 0.05^c^	52.78 ± 0.11^a^	3207 ± 0.92	42.68 ± 0.69^a^
4	48.0 ± 0.20^c^	19.29 ± 0.13b^c^	4.05 ± 0.14^c^	13.3 ± 0.06	7.52 ± 0.13	7.84 ± 0.02^c^	51.62 ± 0.16^a^	3207 ± 0.70	39.67 ± 0.21^b^
6	47.95 ± 0.17^c^	19.28 ± 0.21^d^	3.97 ± 0.06^d^	13.28 ± 0.12	7.52 ± 0.10	8.0 ± 0.12b^c^	49.92 ± 0.06^ab^	3205 ± 0.95	36.36 ± 0.24^b^
8	46.50 ± 0.24^d^	18.99 ± 0.02^d^	3.49 ± 0.02^c^	13.28 ± 0.14	7.52 ± 0.13	10.22 ± 0.03^b^	49.70 ± 0.15^b^	3202 ± 0.65	32.22 ± 0.41^b^
10	45.62 ± 0.19^c^	18.83 ± 0.06^d^	3.33 ± 0.01^c^	13.26 ± 0.15	7.52 ± 0.07	11.44 ± 0.05^b^	49.51 ± 0.12^b^	3202 ± 0.94	26.98 ± 0.21^c^
12	45.21 ± 0.21^e^	18.01 ± 0.17^e^	3.21 ± 0.01^c^	13.22 ± 0.03	7.51 ± 0.06	12.21 ± 0.03^a^	49.01 ± 0.10^b^	3201 ± 0.72	22.41 ± 0.13^c^

The means with different superscripted alphabets are significantly different along the column (*P* < 0.05).

### Effect of polypropylene at freezing temperature on the nutritional composition

That carbohydrate, protein, and fat reduced throughout the storage period from 53.50% to 45.24%, 20.28% to 18.54%, and 4.34% to 2.64%, respectively, as shown in Table2. When food product is exposed to environment above or below its equilibrium point, the protective packages and the barrier level will determine how much the food will be impacted (Lopez 1993). Moisture content increased from 4.38% to 12.54%. The moisture content of mushroom in polypropylene increased more than that observed for mushroom in laminated aluminum foil due to high rate of migration of water vapor from the storage environment to the polypropylene packing material (Barron 2006).

**Table 2:** Proximate composition (%), Minerals and Vitamin (mg/100g) composition of mushroom in polypropylene at freezing temperature

Weeks	CHO	Crude protein	Crude fat	Crude fiber	Ash	Moisture	Ca	K	Vitamin C
0	53.5 ± 0.12^a^	20.28 ± 0.05^a^	4.34 ± 0.14^a^	13.49 ± 0.12	7.67 ± 0.02	4.38 ± 0.02^d^	54.01 ± 0.14^a^	3210 ± 0.60	42.80 ± 0.22^a^
2	50.42 ± 0.12^b^	19.58 ± 0.12^a^	3.92 ± 0.08^ab^	13.45 ± 0.13	7.65 ± 0.11	4.98 ± 0.15^c^	53.98 ± 0.01^a^	3205 ± 0.72	42.78 ± 0.68^a^
4	50.19 ± 0.10^c^	19.40 ± 0.13^b^	3.90 ± 0.14^ab^	1344 ± 0.16	7.65 ± 0.03	5.42 ± 0.12^c^	51.62 ± 0.16^a^	3202 ± 0.70	39.67 ± 0.11^b^
6	49.57 ± 0.12^c^	19.33 ± 0.11^b^	3.72 ± 0.16^b^	13.44 ± 0.22	7.63 ± 0.12	7.31 ± 0.02^b^	51.12 ± 0.16^b^	3201 ± 0.85	36.67 ± 0.14^b^
8	47.45 ± 0.21^c^	18.87 ± 0.02^c^	3.23 ± 0.12^c^	13.43 ± 0.04	7.53 ± 0.13	9.39 ± 013^b^	49.88 ± 0.15^b^	3201 ± 0.55	36.67 ± 0.21^b^
10	46.63 ± 0.14^d^	18.58 ± 0.04^c^	3.10 ± 0.11^c^	13.43 ± 0.05	7.61 ± 0.17	10.65 ± 0.05^a^	49.67 ± 0.02^b^	3201 ± 0.84	28.58 ± 0.11^c^
12	45.24 ± 0.21^d^	18.54 ± 0.12^c^	2.64 ± 0.11^d^	13.43 ± 0.13	7.61 ± 0.16	12.54 ± 0.03^a^	49.01 ± 0.20^b^	3204 ± 0.62	22.54 ± 0.23^c^

The means with different alphabets are significantly different along the column (*P* < 0.05).

### Effect of laminated aluminum foil at freezing temperature on the nutritional composition

The changes in mushroom in laminated aluminum foil at freezing temperature are shown in Table3. Carbohydrate, protein, fat reduced from 53.5% to 47.0%, 20.3% to 17.3%, and 4.3% to 3.3%, respectively. Fiber and ash reduced slightly from 13.5% to13.4% and from 7.7% to 7.6%, respectively, while moisture content increased from 4.4% to 11.4%. Moisture content increased from 4.4% to 11.4% which was better than that of HDPE which might also be as a result of permeability of laminated aluminium foil to gas and moisture (Sethi and Anand 2004)

**Table 3:** Proximate composition (%), Minerals and Vitamin (mg/100 g) composition of mushroom in laminated aluminum foil at freezing temperature

Weeks	CHO	Crude protein	Crude fat	Crude fiber	Ash	Moisture	Ca	K	Vitamin C
0	53.50 ± 0.11^a^	20.28 ± 0.13^a^	4.34 ± 0.12^a^	13.49 ± 0.21	7.67 ± 0.02	4.38 ± 0.03^d^	54.01 ± 0.04^a^	3210 ± 0.42	42.80 ± 0.14^a^
2	51.39 ± 0.06^a^	18.16 ± 0.05^ab^	3.24 ± 0.05^b^	13.450.14	7.60 ± 0.06	6.16 ± 0.10^c^	53.48 ± 0.01^a^	3207 ± 0.87	42.67 ± 0.23^a^
4	50.09 ± 0.24^a^	18.1 ± 0.21^ab^	3.94 ± 0.06^b^	13.44 ± 0.05	7.59 ± 0.01	6.84 ± 0.02^c^	52.92 ± 0.03^a^	3204 ± 0.91	39.67 ± 0.04^b^
6	48.83 ± 0.16^b^	18.05 ± 0.13^b^	3.74 ± 0.12^c^	13.41 ± 0.07	7.58 ± 0.05	8.40 ± 0.21^c^	51.92 ± 0.10^a^	3203 ± 0.84	36.68 ± 0.15^b^
8	47.85 ± 0.12^b^	17.980 ± 12b^c^	3.57 ± 0.20^c^	13.41 ± 0.10	7.58 ± 0.03	9.61 ± 0.03^b^	50.78 ± 0.02^a^	3203 ± 0.74	32.67 ± 0.21^b^
10	47.53 ± 0.02^b^	17.8 ± 0.14^c^	3.32 ± 0.06^d^	13.38 ± 0.08	7.55 ± 0.04	10.42 ± 0.20^b^	49.99 ± 0.03^b^	3202 ± 0.92	28.18 ± 0.10^c^
12	47.01 ± 0.13^b^	17.34 ± 0.13^c^	3.32 ± 0.14^d^	13.36 ± 0.16	7.55 ± 0.01	11.42 ± 0.10^a^	49.99 ± 0.02^b^	2202 ± 0.85	23.74 ± 0.22^c^

The means with different alphabets are significantly different along the column (*P* < 0.05).

### Effect of vacuum-packaged mushroom at freezing temperature on the nutritional composition

Calcium and vitamin C reduced from 54.0 to 50 mg/100 g and 42.8 to 23.7 mg/100. Table4 shows the change in the nutritional composition of dried mushroom packaged in HDPE and packaged under vacuum at freezing temperature. Carbohydrate, protein, fat reduced slightly from 53.5% to 48.5%, 20.3% to 19.1%, and from 4.3% to 3.4%, respectively, while fiber and ash also reduced insignificantly from 13.5% to 13.3% and from 7.7% to 7.6% and moisture content increased from 4.4% to 6.6%. The maximum nutrient retention capacity was recorded for vacuum packaging which shows slight difference throughout the storage period and this follows (Chang and Miles 2004) findings that vacuum packaging is process of removing air around a food product and then sealing that product in an impermeable package. Removing the air that surrounds food inhibits growth of bacteria, mold, and yeast, because these and other spoilage microorganisms need oxygen to grow. Once moist air is removed and the pouch is sealed, oxygen levels continue to drop while carbon dioxide levels increase. The low oxygen, high carbon dioxide environment significantly reduces the growth of normal spoilage organisms, allowing longer nutritional retention.

**Table 4:** Proximate composition (%), Minerals and Vitamin (mg/100 g) composition of mushroom in HDPE packaged under vacuum at freezing temperature

Weeks	CHO	Crude protein	Crude fat	Crude fiber	Ash	Moisture	Ca	K	Vitamin C
0	53.5 ± 0.03	20.28 ± 0.06	4.34 ± 0.01^a^	13.49 ± 0.03^a^	7.67 ± 0.02	4.38 ± 0.01^c^	54.01 ± 0.02	3210 ± 0.74	42.84 ± 0.02^a^
2	50.0 ± 0.23	19.99 ± 0.13	3.86 ± 0.02^a^	13.44 ± 0.12^a^	7.67 ± 0.13	5.04 ± 0.12^c^	53.78 ± 0.01	3207 ± 0.89	42.47 ± 0.13^a^
4	49.57 ± 0.12	19.92 ± 0.12	3.84 ± 0.01^a^	13.41 ± 0.01^ab^	7.64 ± 0.01	5.62 ± 0.11^b^	53.86 ± 0.10	3206 ± 0.60	38.69 ± 0.10^b^
6	49.2 ± 0.06	19.88 ± 0.14	3.88 ± 0.10^a^	13.41 ± 0.20^ab^	7.63 ± 0.02	6.0 ± 0.02^b^	53.84 ± 0.12	3206 ± 0.89	36.59 ± 0.03^b^
8	49.22 ± 0.12	19.71 ± 0.03	3.62 ± 0.03^a^	14.38 ± 0.04^b^	7.63 ± 0.04	6.44 ± 0.13^b^	53.84 ± 0.01	3205 ± 0.74	32.25 ± 0.12^b^
10	49.15 ± 0.02	19.57 ± 0.21	3.59 ± 0.12^b^	13.37 ± 0.01^b^	7.62 ± 0.03	6.50 ± 0.12^b^	53.58 ± 0.09	3205 ± 0.70	29.90 ± 0.10^c^
12	48.50 ± 0.23	19.14 ± 0.10	3.42 ± 0.03^b^	13.4 ± 0.02^b^	7.59 ± 0.10	6.64 ± 0.11^a^	53.23 ± 0.10	3184 ± 0.72	26.24 ± 0.02^c^

The means with different alphabets are significantly different along the column (*P* < 0.05).

### Microbiological assessment of dried mushroom in different packaging materials

Table5 shows the microbial assessment of dried mushroom in different packaging materials. Dried mushroom packaged in HDPE at freezing temperature had mean levels of 3.2 and 1.1 log cfu/g for total aerobic count and Pseudomonal count, respectively. Dried mushroom in laminated aluminum foil at freezing temperature showed the mean counts of 2.8, 1.5, and 0.5 log cfu/g for total aerobic count*, Pseudomonal count*, *and Staphylococcal count,* respectively, while vacuum-packaged dried mushroom at freezing temperature gave mean count 2.3, 0.5 and 0.4 log cfu/g, respectively, for *total aerobic count, Pseudomonas*, *and Staphylococcal* count. *Salmonella* and *Coliform* spp. were not detected in all the samples analyzed. The safety of food is based on the International Food Standards; they are classified as A, B, C, and D according to the European Commission Regulation (EC) No 2073/2005. Mushroom under Class A foods are regarded as satisfactory, Class B foods are regarded as less than satisfactory but can still be acceptable for consumption; Class C are categorized as unsatisfactory, while class D imply that the microbiological status of the food sample is unacceptable as it contains unacceptable levels of specific pathogens that are potentially hazardous to the consumer.

**Table 5:** Microbiological assessment of dried mushroom in different packaging materials at the 12th week

Packaging materials	Total aerobic count (log cfu/g)	*Pseudomonas* spp. (log cfu/g)	*Salmonella* spp. (log cfu/g)	Coliform count (log cfu/g)	Staphylococcal count (log cfu/g)
HDPE	3.24 ± 0.03^b^	1.13 ± 0.02^a^	ND	ND	ND
PP	3.76 ± 0.04^a^	1.09 ± 0.01^b^	ND	ND	0.54 ± 0.02^b^
LAF	2.75 ± 0.01^a^	1.54 ± 0.03^a^	ND	ND	0.47 ± 0.04^b^
HDPEV	2.31 ± 0.03^a^	0.46 ± 0.01^b^	ND	ND	0.37 ± 0.01^a^

The means with different superscripted alphabets are significantly different along the column (*P* < 0.05).

Based on the International Food Standard, the total aerobic count, *Pseudomonas* spp., *total coliform, Salmonella* spp.*, Staphylococcal* spp. count in the mushroom sample, must be <3 log cfu/g, <1.3 log cfu/g, <1.3 log cfu/g, <1.30 log cfu/g, <1.3 log cfu/g, respectively, to be classified as satisfactory according to the European Commission Regulation (EC No 2073/2005). The total aerobic count ranged from 2.31 to 3.76 log cfu/g with dried mushroom packaged in high density polyethylene at ambient temperature having the highest value meanwhile the lowest value was observed in mushroom packaged under vacuum at freezing temperature with the value of 2.3 log cfu/g which is less than the 3.0 log cfu/g thus, should be proposed as satisfactory by the European Commission Regulation (EC) No 2073/2005. Salmonellae are pathogens which are one of the most common causes of bacterial food poisoning. Salmonellosis (i.e., the disease caused by *Salmonella enterica*) is principally a food-borne disease, although other possible routes of transmission include contact with infected animals or their fecal material, person to person spread, and nosocomial infection. Salmonellae reside in the intestinal tract of infected animals and humans and are shed in the feces. Foods subject to fecal contamination (e.g., agricultural products, meat) are among those which have been implicated as vehicles in the transmission of this pathogen to humans. Contamination of agricultural products can occur at any stage during cultivation, harvest, or postharvest. In this research, *Salmonella* spp. was not detected in all the packaged samples analyzed according to Table5. *E*. *coli* is an enteric organism. Most strains of *E*. *coli* are harmless; however, several are known to be pathogenic. *E*. *coli* is often used as an indicator of fecal contamination in food. In this research, *Coliform* spp. were not also detected in all the packaged sample analyzed. *S*. *aureus* is a pathogenic bacterium which is a common cause of food poisoning. Staphylococcal food poisoning is caused by ingestion of a heat stable toxin formed by *S*. *aureus* in the food. Both the onset and the severity of the symptoms depend on the susceptibility of the person and the amount of toxin consumed. The main symptoms include abdominal cramps, vomiting, and diarrhea. Food handlers are commonly implicated in the transmission of this pathogen to food (it is estimated that up to 50% of humans are carriers of this bacterium on their skin, nose, and throat). The value of *S. aureus in* mushroom packaged under vacuum is 0.4 log cfu/g which is satisfactory according to according to European Commission Regulation (EC) No 2073/2005.

## Conclusion

Dried mushroom packaged under vacuum and stored at freezing conditions plays a relatively important part in extending shelf life of dried mushroom. It is effective in controlling microorganism and enhancing wholesomeness of packaged mushroom over an extended period of time.
